# Impact of nuclear YAP1 expression in residual cancer after neoadjuvant chemohormonal therapy with docetaxel for high-risk localized prostate cancer

**DOI:** 10.1186/s12885-020-06844-y

**Published:** 2020-04-15

**Authors:** Yoshinori Matsuda, Shintaro Narita, Taketoshi Nara, Huang Mingguo, Hiromi Sato, Atsushi Koizumi, Sohei Kanda, Kazuyuki Numakura, Mitsuru Saito, Takamitsu Inoue, Yuko Hiroshima, Hiroshi Nanjo, Shigeru Satoh, Norihiko Tsuchiya, Tomonori Habuchi

**Affiliations:** 1 Department of Urology, Akita University School of Medicine, 1-1-1 Hondo, Akita, 010-8543 Japan; 2 Department of Pathology, Akita University Hospital, Akita, Japan; 3 Center for Kidney Disease and Transplantation, Akita University Hospital, Akita, Japan; 4 Department of Urology, Yamagata University School of Medicine, Akita, Japan

**Keywords:** YAP1, Residual cancer, Neoadjuvant chemohormonal therapy, Docetaxel, Prostate cancer, High-risk

## Abstract

**Background:**

Although docetaxel-based chemohormonal therapy (CHT) is one of the standard treatments for castration-resistant prostate cancer (CRPC), pertinent biomarkers and precise mechanisms involved in the resistance for CHT for CRPC remain unknown. We investigated the relationship between chemohormonal resistance and the expression of steroid receptors and Hippo pathway proteins using a docetaxel-resistant prostate cancer (PCa) cell line and human PCa tissues in patients who underwent surgery with and without neoadjuvant therapy.

**Methods:**

A docetaxel-resistant subline (22Rv1-DR) was generated to assess Hippo pathway protein expression and the effect of YAP1 inhibition on cellular characteristics. A tissue microarray with 203 cores from 70 high-risk localized PCa tissues was performed to assess steroid receptor and Hippo pathway protein expressions.

**Results:**

Nuclear YAP (nYAP) expression was higher in 22RV-1-DR than in parental 22Rv-1 and YAP1 knockdown suppressed cell proliferation of 22Rv1-DR. Steroid receptor and Hippo pathway protein expressions varied among three different neoadjuvant groups, and nYAP1 expression was the highest in the CHT group. The patients with high nYAP in residual cancer after neoadjuvant CHT had a significantly higher biochemical recurrence (BCR) rate than those with low nYAP1. On multivariate analysis, the high nYAP1 was an independent prognostic factor for BCR.

**Conclusions:**

nYAP expression is a potential biomarker in high-risk patients treated with docetaxel-based CHT. Steroid receptors and Hippo pathway proteins may play a role in the chemohormonal resistance in advanced PCa.

## Background

Prostate cancer (PCa) is one of the most common cancers and the major leading cause of cancer-related death in men worldwide [1]. A recent epidemiological study showed that PCa had the highest incidence of cancer for men in 92 countries and the leading cause of cancer deaths for men in 48 countries [2]. Although androgen deprivation therapy (ADT) is a mainstay treatment for advanced PCa, eventually all patients develop conventional ADT-resistant cancer, known as castration-resistant prostate cancer (CRPC). After two large phase III randomized trials demonstrated that docetaxel-based chemotherapy improved the survival of patients with CRPC [3, 4], docetaxel-based chemotherapy is one of the standard treatments for CRPC. However, a significant heterogeneity was found in the response of patients to ADT and/or chemotherapy, and most patients become refractory due to the development of drug resistance. Therefore, the identification of key regulators of resistance to both hormonal therapy and chemotherapy in patients with advanced PCa is warranted.

Recent randomized phase III trials have shown that the combination therapy of ADT plus docetaxel for newly diagnosed metastatic hormone-naive PCa provides significant survival benefits compared with ADT alone [5, 6]. However, whether the same extent of benefit is obtained if these therapies are applied at an earlier stage, such as high-risk localized PCa, remains controversial. We previously reported the outcomes of neoadjuvant chemohormonal therapy (CHT) combined with ADT, docetaxel, and estramustine phosphate, followed by radical prostatectomy (RP) [7, 8] . In these studies, we found the limitation of the effect of CHT in patients with high-risk localized PCa, therefore indicating the importance of elucidating the molecular mechanisms underlying the resistance to docetaxel–CHT to overcome resistance and improve the outcome for the treatment of high-risk localized PCa.

Numerous previous studies have hypothesized that mechanisms and pathways are implicated in the development of resistance to ADT and docetaxel in PCa [9–11]. Both androgen receptor (AR)-dependent and -independent mechanisms were considered to be associated with resistance for PCa treatment [12]. Based on tissue biomarker analyses, the expression of AR signaling, steroid synthesis pathway, intracellular signaling, stromal–epithelial interaction, hedgehog signaling, and angiogenesis pathway were considered to be biomarkers for drug resistance and prognoses in patients treated with neoadjuvant chemotherapy and CHT, followed by RP [13, 14].

The Hippo pathway is known to regulate tissue homeostasis, organ size, and tumorigenesis, and exerts a significant impact on cancer development by modulating cell proliferation, apoptosis, and stemness in response to a wide range of extra- and intracellular signals, including cell–cell contact, cell polarity, mechanical cues, ligands of G-protein-coupled receptors, and cellular energy status [15]. Moreover, increased nuclear localization and higher transcriptional activities of YAP/TAZ, main transcriptional regulators in the Hippo pathway, have been observed in therapy-resistant malignancies [16]. With regard to the relationship between Hippo signaling and PCa, a recent study showed that YAP1 and AR are co-localized and interact with each other predominantly within the cell nuclei by an androgen-dependent mechanism in hormone-naive PCa and an androgen-independent mechanism in CRPC cells [17]. Moreover, recent studies have shown that the Hippo pathway is involved in docetaxel-resistance of prostate cancer [18, 19]. However, the impact of Hippo pathway proteins on ADT- and chemotherapy-resistant PCa progression remains unknown. In particular, no study has evaluated the expression levels of the Hippo pathway proteins in PCa patients with and without neoadjuvant treatment.

In this study, we investigated Hippo pathway protein expression and the effect of YAP1 inhibition on cellular characteristics such as proliferation, apoptosis and the cell cycle in a docetaxel-resistant PCa cell line. Moreover, the tissue expression of candidate biomarkers, including Hippo pathway proteins and steroid receptors, was assessed using human PCa tissues in patients who underwent RP with and without neoadjuvant therapy to identify tissue biomarkers in patients with high-risk PCa treated with CHT and to explore novel targets for chemohormonal resistance for advanced PCa.

## Methods

### Cell lines

Human PCa 22Rv1 cells were obtained from the American Type Cell Culture Collection (Manassas, VA, USA). The cells were authenticated with short-tandem repeat analysis (Bio-Synthesis, Lewisville, TX, USA). The docetaxel-resistant subline of 22Rv1 cell (22Rv1-DR), which has a characteristic of androgen- and ligand-independent growth [20, 21], was established in the presence of increasing concentrations of docetaxel (Sigma-Aldrich, St. Louis, MO, USA) up to the final concentration of 5 nM, which is the IC_50_ concentration in our pilot study. Development of the drug-resistant cell line took ≥4 months and further studies using sublines cultured for ≥4 months that were based on the results of MDR1 expression in the cells were performed. The cells were cultured in RPMI 1640 medium (Invitrogen, Carlsbad, CA, USA) containing 10% fetal bovine serum (FBS) in a 5% CO_2_ humidified incubator at 37 °C and passaged for 3–4 days in a fresh medium to achieve approximately 80% confluency. SiYAP1 (SI02662954) and negative control siRNA (AllStars Negative Control siRNA) were purchased from Qiagen (Valencia, CA). Transfections of siRNAs were performed by using Lipofectamine 3000 (Invitrogen) according to the manufacturer’s procedure.

### Cell proliferation assay

A non-radioactive MTT-based cell proliferation assay kit (Roche, Switzerland) was used based on the manufacturer’s instructions. The proliferation assays were performed in triplicate. A total of 1.0 × 10^4^ 22Rv1 cells were seeded into each well of a 24-well plate and incubated for 72 h with fresh media containing 10% FBS. Absorbance was measured using an enzyme-linked immunosorbent assay reader (BIO RAD, Hercules, CA, USA).

### Apoptosis and cell cycle analyses

Equal numbers of 22Rv1-DR cells (5 × 10^5^) were plated into a six-well plate. Three day later, the cells were treated with 25 nM siRNAs. For apoptosis and cell cycle analyses, the cells were analyzed by using a Cycletest Plus DNA Reagent kit (BD Biosciences, Franklin Lakes, NJ, USA) according to the manufacturer’s protocol. The rate of apoptosis and fraction of each cell cycle phase (subG0,G0-G1, S, G2) were examined by using a FACSCalibur flow cytometry system (BD Biosciences, Franklin Lakes, NJ, USA).

### Western blot analysis

Total proteins were isolated using complete Lysis-M buffer (Roche). The protein concentration was measured using the ND-1000 method (Thermo Fisher Scientific). Equal amounts of protein lysates were separated by sodium dodecyl sulfate polyacrylamide gel electrophoresis and transferred using the iBot® Blotting System (Invitrogen). The membranes were blocked for 1 h at room temperature with a buffer containing 2% bovine serum albumin in Tris-buffered saline with 0.1% Tween-20. The membranes were incubated overnight in the diluted antibodies and blocked with secondary immunoglobulin G (IgG) antibody for 1 h. Specific proteins were detected using the ECL prime western blotting detection reagent (Amersham Biosciences, Buckinghamshire, UK). The monoclonal/polyclonal antibodies, including PARP, MDR1, YAP1 (= YAP, YAP65), p-YAP, TAZ, β-actin, Lamin A/C (Cell Signaling Technology), MOB1B (= MOB4A, Abgent), and TEAD1 (BD Biosciences, city, US state, country), were used. Nuclear and cytoplasmic fractions were prepared using the NE-PER Nuclear and Cytoplasmic Extraction reagents based on the manufacturer’s instruction (Thermo Fisher Scientific).

### Patients

The patients were prospectively enrolled in the phase II study to assess the impact of CHT with ADT, docetaxel, and estramustine phosphate, followed by RP in patients with high-risk PCa between 2006 and 2016 at our institution [7, 8]. Eligible patients had histopathologically confirmed localized, high-risk PCa and were candidates for RP at our hospital as reported previously [7, 8]. The patients were excluded if they had received prior therapy for PCa, prior invasive malignancy, any serious comorbidity, or an Eastern Cooperative Oncology Group performance status of ≥2. All the patients provided written informed consent, and the study was approved by the institutional review board of Akita University (Ethical Approval No. 1341). A “high-risk” disease was defined as any of the following conditions: ≥cT3, preoperative PSA level of ≥15 ng/mL, and/or a Gleason pattern of 5 in primary and/or secondary. Supplementary Table 1 describes the patient characteristics in the CHT group. To establish the tissue microarray (TMA), we also included patients with PCa with our high-risk criteria who underwent RP without any neoadjuvant therapy (no neoadjuvant: NNA) and with neoadjuvant hormonal therapy (NHT) for > 3 months in the same period.

### Treatment regimen

Supplementary Figure 1 shows the schedule of CHT at our institution [8]. Briefly, the CHT protocol involved combined androgen blockade with 11.25 mg leuprorelin or goserelin subcutaneously once every 3 months and 81 mg bicalutamide orally for the first 12 weeks. Docetaxel at a dose of 30 mg/m^2^ was administered intravenously, with 560 mg of estramustine phosphate orally for 6 consecutive weeks. The clinical outcomes of the study have been reported in previous literature [7, 8].

### Tissue microarrays and immunohistochemical analyses

A TMA was constructed at the Pathology Institute, Toyama, Japan, using paraffin blocks of primary prostatectomy tissues from the patients treated at our institute. Briefly, two independent pathologists (H.N. and Y.H.) blinded to the identity of the patient associated with each tissue analyzed the hematoxylin and eosin–stained sections of each paraffin block, and the area of residual PCa within each section was identified. Triplicated cores measuring 0.6 mm in diameter were collected randomly from the cancerous areas and transplanted to the TMA. Tissues were excluded if no residual cancerous regions in the prostate (pT0) were found. As a result, a TMA had 210 cores from 70 high-risk patients with localized PCa who underwent RP with NNA (*n* = 15), with NHT (*n* = 11), or with CHT (*n* = 44) (Supplementary Fig. 2). In establishing the TMA, seven cores were excluded as part of the TMA slide. Therefore, the final number of cores embedded in the TMA was 203. The expression of six candidate biomarkers, including steroid receptors (AR, glucocorticoid receptor [GR], progesterone receptor [PR], and estrogen receptor alpha [ERα]), and Hippo pathway proteins (YAP1 and MOB4A), were statistically assessed using immunohistochemistry (IHC). Supplementary Table 2 lists the antibodies used in this study. The IgG isotype controls were used as a negative control. To assess the expression of tissue markers, the intensity of IHC staining was scored and stratified into four groups: negative (0), weak (1), moderate (2), and strong (3). The area of IHC staining was also stratified into five groups: 0% (0), < 25% (1), < 50% (2), < 75% (3), and 100% (4). Subsequently, the intensity, area, and immunoreactivity scores, which were determined by multiplying the intensity and area [22], were evaluated. Cytoplasmic AR and GR expressions were indicated as negative or positive. The cores were excluded from scoring if determining the scores was difficult due to peeling off of a part of the tissue from the slide and if there were no adequate residual cancer epithelial cells in the section. The differential expression of the six biomarkers between the three groups and the impact of expression scores on biochemical recurrence (BCR) in the CHT group were statistically assessed.

### Statistical analyses

Immunohistochemical scores were reported as means ± standard errors. Differences in the scores of immunohistochemical staining among the three groups were evaluated using the chi-squared test and analysis of variance. Differences of expression levels of biomarkers in the NNA and NHT groups were statistically compared with those in the CHT group using the chi-squared test and the Mann–Whitney U test for categorical and continuous variables, respectively. The date of BCR was defined as that when the serum PSA level exceeded 0.2 ng/mL or when adjuvant or salvage therapy was initiated even if PSA did not exceed 0.2 ng/mL. BCR-free survival was calculated using the Kaplan–Meier method with log-rank tests for between-group comparisons. Independent prognostic factors were identified by univariate analysis (i.e., patient age, baseline PSA level, Gleason score at diagnosis, CHT completion, extended lymph node dissection, pathological T stage, pathological N stage, positive surgical margin, and scores of immunostaining of the six biomarkers). Significant preoperative variables in univariate analyses (*p* < 0.05) were included in multivariable analyses, which were performed using the Cox proportional hazards regression model. SPSS, version 24.0® (SPSS Inc., Chicago, IL, USA) was used for statistical analysis, and all *p*-values were two-sided and considered significant when < 0.05.

## Results

### Establishment of docetaxel-resistant 22Rv1 sublines and hippo pathway protein expression

**Fig. 1:**
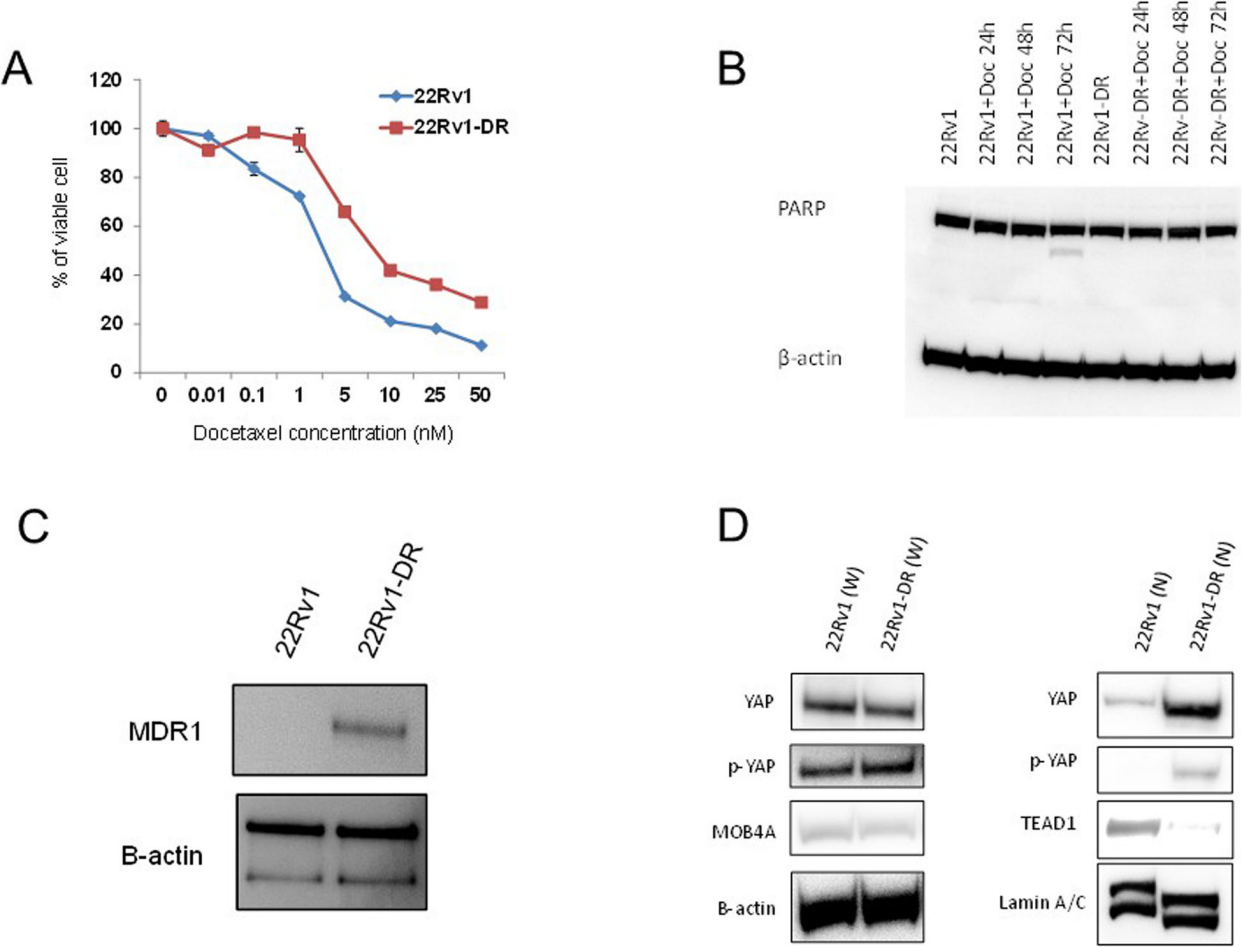
Establishment of docetaxel-resistant 22Rv1 subclones and Hippo pathway protein expression. **a** Cell proliferation of 22Rv1 and 22Rv1-DR cells. A total of 1.0 × 104 cells were seeded into each well of a 24-well plate and incubated for 72 h containing 10% FBS. Cell proliferation was determined using a non-radioactive MTT-based cell proliferation assay kit. **b** PARP protein expression in the 22Rv1 cell lines with different docetaxel concentrations and timing were measured by western blotting. β-actin was used as a loading control. **c** MDR-1 protein expression in the 22Rv1 and 22Rv1-DR cell lines was measured by western blotting. 22Rv1-DR cells were cultured for 4 months in medium with docetaxel. β-actin was used as a loading control. **d** Whole and nuclear expression of Hippo pathway proteins.β-actin was used as a loading control for whole cell lysates, whereas Lamin A/C was used as a loading control for nuclear lysates. Blotting signals were captured using CS Analyzer 3.0 software (ATTO). The blots in the figure were cropped, but the polyacrylamide gels were run under the same experimental conditions. The original gel images were presented in Supplementary Figure 3, 4 and 5

First, we established the 22Rv1-DR cell subline, which was resistant to various concentrations of docetaxel, compared with the parental 22Rv1 cell (Fig. 1a). The IC_50_ at 72 h in the 22Rv1-DR cells was significantly higher than that in the parental 22Rv1 cells (7.92 vs 2.39 nmol/L, respectively, *p* = 0.002). To confirm the resistance to apoptosis after docetaxel treatment, the PARP expression in parental 22Rv1, 22Rv1-DR, and two cell lines after docetaxel treatment with different durations of exposure was determined (Fig. 1b, sFig.3). The cleaved PARP was observed in the parental 22Rv1 treated for 72 h with 5 nmol/L of docetaxel, whereas no cleaved PARP expression was shown in the 22Rv1-DR up to 72 h after treatment of docetaxel (Fig. 1b, sFig.3). The results confirmed that the 22Rv1-DR subline was resistant to apoptosis induced by docetaxel treatment. Consistent with the previous reports [23, 24], the expression of MDR-1, which is a robust drug pump, was markedly higher in the 22Rv1-DR sublines than that in the parental 22Rv1 (Fig. 1c, sFig.4). Using the cell lines, the expression and activation of Hippo pathway proteins, including YAP1, TAZ, MOB4A, and TEAD1, were subsequently compared in 22Rv1-DR and 22Rv1 cells (Fig. 1d, sFig.5). These four proteins are known to be key regulators of Hippo pathway signaling [15]. With regard to Hippo pathway protein expression in the whole lysates, no difference in expression was found in YAP, p-YAP, and MOB4A between the parental 22Rv1 and 22RV1-DR cells (Fig. 1d). In the nuclear fraction of lysates, nuclear YAP1 (nYAP1) and p-YAP1 expressions in 22Rv1-DR were markedly higher than those in 22Rv1, whereas nuclear TEAD1 expression was lower in 22Rv1-DR (Fig. 1d, sFig.5). Consistent with the previous study [25], TAZ was not expressed in 22Rv1 and its sublines (data not shown), suggesting that nYAP1 expression and activation were associated with chemotherapy resistance in the 22Rv1 PCa cell line and that nYAP1 expression and activation have a potential to be associated with chemohormonal resistance for PCa.

### Effect of YAP1 knockdown on cellular characteristics in the 22Rv1-DR cells

**Fig. 2:**
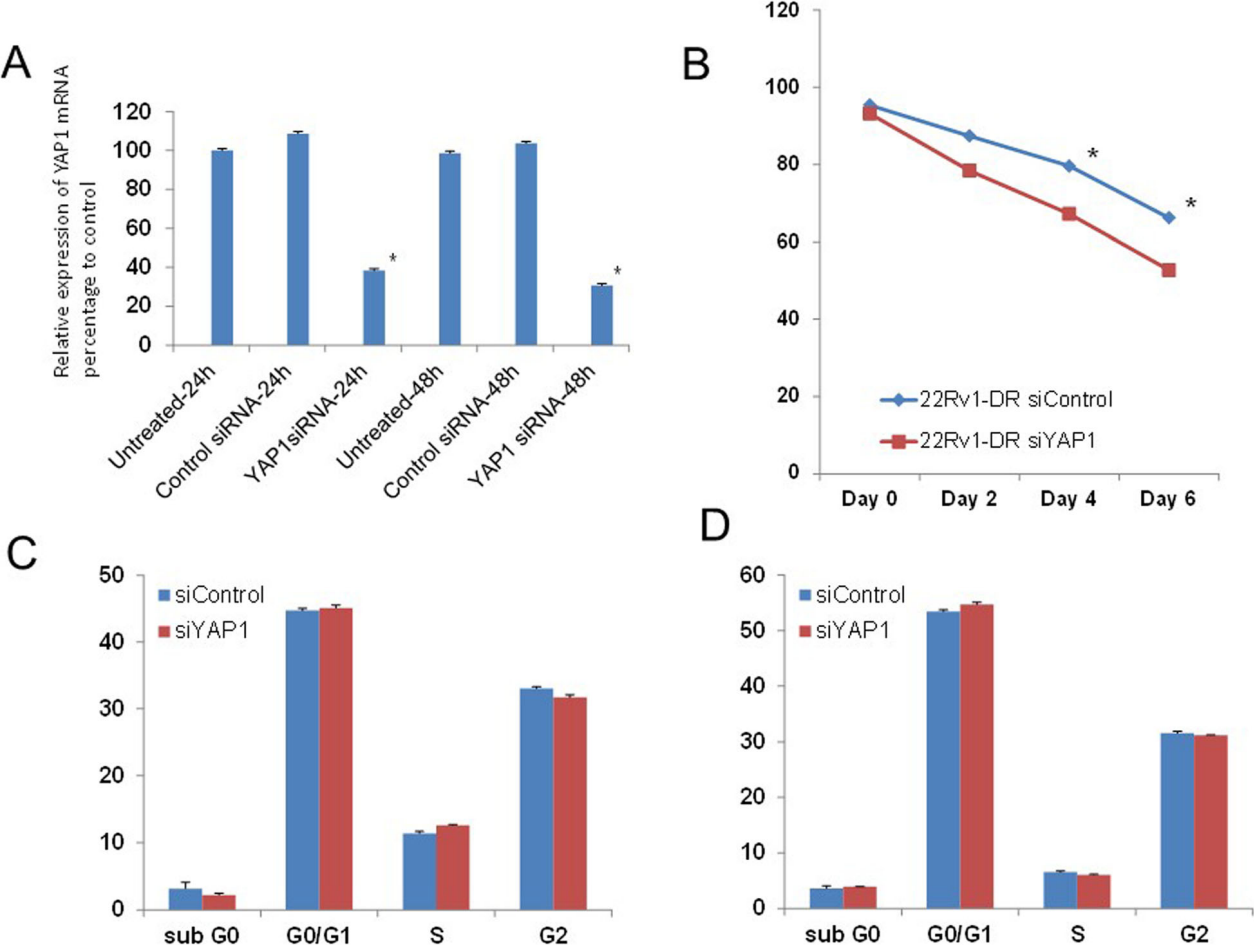
The effects of YAP1 knockdown on cell proliferation, induction of apoptosis and modulation of the cell cycle phase. **a** 22Rv1-DR cells were treated with 25 nM siYAP1 or siControl for 24–48 h. The day after treatment, total cellular RNA was extracted, and YAP1 mRNA expression was analyzed by quantitative RT-PCR. The YAP1 mRNA levels of the cells treated with siRNAs were normalized to the mRNA levels of the untreated 22RV-1-DR cells. **p < 0.05. **b** 22Rv1-DR cells were treated with 25 nM of siYAP1 or siControl for ≤6 days. Cell viability was determined by MTT-based cell proliferation assay. The cell number after siRNA induction was compared with that of the untreated 22Rv1-DR cells. *p < 0.05. **c**, **d** 22Rv1-DR cells were treated with 25 nM siRNAs for 24 h (**c**) and 48 h (**d**), and then harvested for flow cytometric analyses. The fractions of each cell cycle phase were compared between the cells treated with siYAP1 and those treated with control siRNA

Next, the effects of YAP1 knockdown on cell proliferation, apoptosis and the cell cycle in established 22Rv1-DR cells were investigated. In the 22Rv1-DR cells, siYAP1 significantly suppressed the level of *YAP1* mRNA in a time-dependent manner (Fig. 2a). Figure 2b showed significant reduction of cell proliferation of 22Rv1-DR cells after treatment with YAP1 siRNA relative to that after treatment with control siRNA at 4 and 6 days after transfection of siRNAs (*p* = 0.038, *p* = 0.049, respectively,). Regarding induction of apoptosis, there was no significant difference in the fraction of 22Rv1-DR cells undergoing apoptosis (sub G1-G0 fraction) between the cells treated with siYAP1 and those treated with the control siRNA at 24 and 48 h after transfection (*p* = 0.439, *p* = 0.613, Fig. 2c and d). Moreover, there were no significant differences in the percentages of 22Rv1-DR cells in the G0/G1, S and G2 fractions between the cells treated with siYAP1 and those treated with control siRNA at 24 and 48 h after transfection (Fig. 2c and d). These results suggested that YAP1 knockdown inhibits cell growth of the 22Rv1-DR cells without induction of apoptosis and modulation of cell cycle arrest.

### Expression of tissue biomarkers in human prostate tissues among three different neoadjuvant treatment groups

**Fig. 3:**
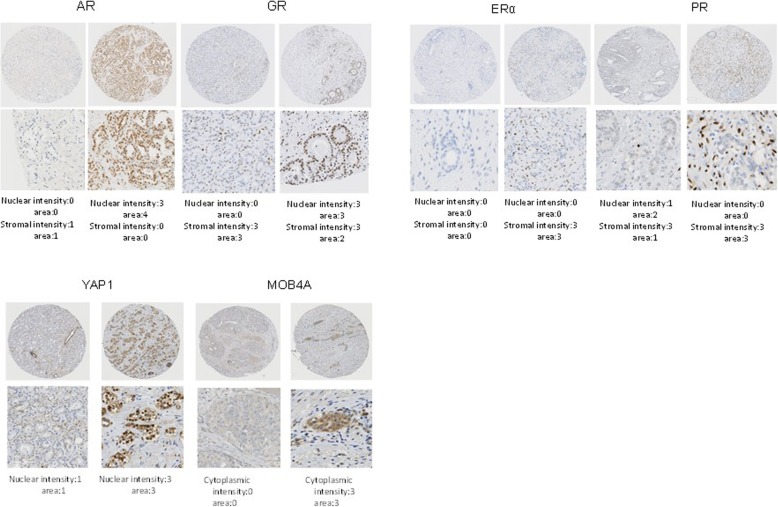
Representative immunohistochemical staining patterns for six biomarkers, including steroid receptors and Hippo pathway proteins in a TMA. For each marker, representative positive and negative areas are described

**Fig. 4:**
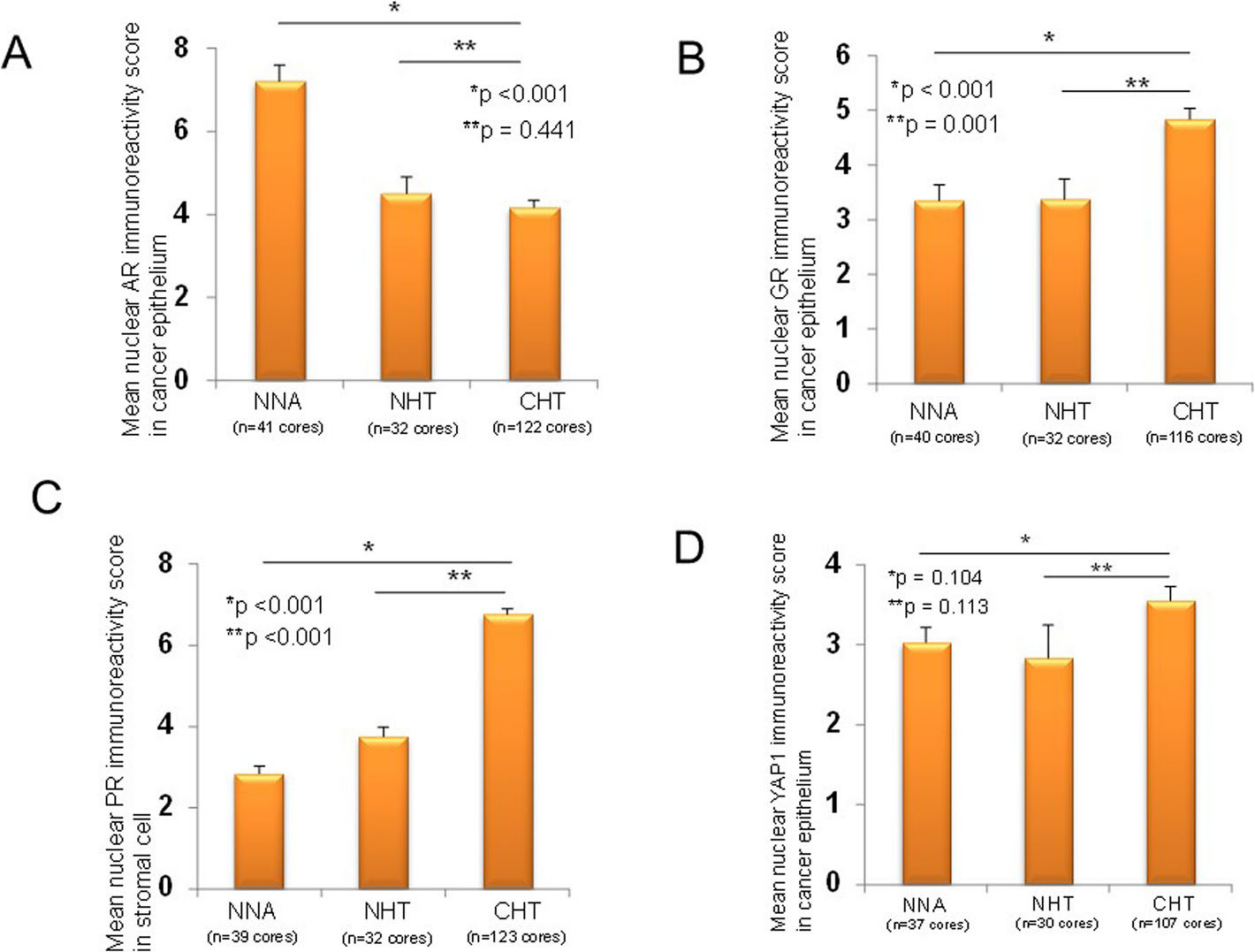
The expression of tissue biomarkers among three different neoadjuvant treatment groups. The mean immunoreactivity score in cancer and stromal cells among the three groups, including NNA, NHT, and CHT, are described. The statistical differences of the mean immunoreactivity score of NHT and CHT compared with NNA were statistically evaluated. *p < 0.05. Nuclear expression of **a** AR in residual cancer cells, **b** GR in residual cancer cells, **c** PR in stromal cells, and **d** YAP1 in residual cancer cells

Subsequently, the difference of expression of six candidate tissue biomarkers, including the Hippo pathway proteins (YAP1 and MOB4A) with steroid receptors (AR, GR, PR, and ERα), which were known to play a key role in PCa progression and resistance [26–28], among the three groups with different neoadjuvant treatments by the IHC analyses was investigated. AR and GR expressions were observed in the nucleus and cytoplasm of residual cancer cells and stromal cells in prostate tissues (Fig. 3), whereas ERα and PR expressions were expressed mainly in the stromal cells of prostate tissues (Fig. 3). With regard to the Hippo pathway proteins, YAP1 was expressed in both the nucleus and the cytoplasm, whereas MOB4A was expressed mainly in the cytoplasm (Fig. 3). Using core-based scoring analyses, AR, GR, PR, ERα, YAP1, and MOB4A expressions among the three groups with various neoadjuvant settings were compared (Table 1). The mean nuclear AR (nAR) immunoreactivity score in residual cancer cells in the CHT group was significantly lower than that in the NNA group (4.16 ± 0.18 vs. 7.20 ± 0.39, respectively, *p* < 0.001), whereas no difference of the nAR immunoreactivity score was found between the NHT and CHT groups (4.16 ± 0.18 vs. 4.50 ± 0.40, respectively, *p* = 0.441, Fig. 4a). The mean nuclear GR imunoreactivity score in the residual cancer cells in the CHT group was significantly higher than that in the NNA (4.83 ± 0.20 vs. 3.35 ± 0.29, respectively, *p* < 0.001) and NHT groups (4.83 ± 0.20 vs. 3.38 ± 0.37, respectively, *p* = 0.001, Fig. 4b). The mean nuclear PR immunoreactivity score in stromal cells in the CHT group was significantly higher than that in the NNA (6.76 ± 0.14 vs. 2.85 ± 0.19, respectively, *p* < 0.001) and NHT groups (6.76 ± 0.14 vs. 3.75 ± 0.24, respectively, *p* < 0.001, Fig. 4c). With regard to Hippo pathway protein expression in the TMA, the mean nYAP area and intensity in the residual cancer cells in the CHT group were significantly higher than those in the NHT group (1.76 ± 0.06 vs. 1.27 ± 0.15, *p* = 0.002; and 1.78 ± 0.05 vs. 1.27 ± 0.15, *p* = 0.002, respectively). The mean cytoplasmic YAP1 immunoreactivity score in residual cancer cells in the CHT group was significantly higher than that in the NNA (4.71 ± 0.20 vs. 2.57 ± 0.26, respectively, *p* < 0.001) and NHT groups (4.71 ± 0.20 vs. 2.90 ± 0.32, respectively, *p* < 0.001). The nYAP1 immunoreactivity score of residual cancer cells in the CHT group was the highest, although no statistical differences were found among the three groups (Fig. 4d). The mean immunoreactivity score of cytoplasmic MOB4A in the CHT group was significantly higher than that in the NNA (4.09 ± 0.13 vs. 2.93 ± 0.24, respectively, *p* < 0.001) and NHT groups (4.09 ± 0.13 vs. 3.20 ± 0.31, respectively, *p* = 0.004). Taken together, the expression changes of the six biomarkers in PCa tissues after the neoadjuvant treatments followed by RP varied. However, several markers, including nuclear GR, stromal PR, nYAP1, and cytoplasmic MOB4A, were upregulated in the CHT group, whereas nAR was downregulated after neoadjuvant treatments with androgen deprivation and/or chemotherapy.

**Table 1:** Expression levels of six potential biomarkers in patients with high-risk prostate cancer who underwent radical prostatectomy with and without neoadjuvant treatment

				NNA	NHT	CHT	*p*-value	*p* value (NNA vs. CHT)	*p* value (NHT vs. CHT)
AR	Epithelial cells	Nuclear	Area	3.01 ± 0.10	2.06 ± 0.13	2.07 ± 0.0.06	< 0.001	< 0.001	0.984
			Intensity	2.27 ± 0.08	1.94 ± 0.12	1.79 ± 0.05	< 0.001	< 0.001	0.208
			Immunoreactivity score	7.20 ± 0.39	4.50 ± 0.40	4.16 ± 0.18	< 0.001	< 0.001	0.441
		Cytoplasmic	Potitive/Negative (% of positive)	60/22 (73.2%)	48/16 (75.0%)	82/162 (33.6%)	< 0.001	< 0.001	< 0.001
	Stromal cells		Area	1.21 ± 0.65	1.72 ± 0.81	1.55 ± 0.64	< 0.001	< 0.001	0.077
			Intensity	1.08 ± 0.06	1.50 ± 0.08	1.33 ± 0.04	< 0.001	< 0.001	0.034
			Immunoreactivity score	1.54 ± 0.14	2.94 ± 0.24	2.26 ± 0.10	< 0.001	< 0.001	0.010
GR	Epithelial cells	Nuclear	Area	1.80 ± 0.10	1.69 ± 0.13	2.36 ± 0.05	< 0.001	< 0.001	< 0.001
			Intensity	1.55 ± 0.10	1.66 ± 0.12	1.88 ± 0.06	0.009	0.004	0.076
			Immunoreactivity score	3.35 ± 0.29	3.38 ± 0.37	4.83 ± 0.20	< 0.001	< 0.001	0.001
		Cytoplasmic	Potitive/Negative (% of positive)	0/80 (0%)	0/64 (0%)	38/196 (16.2%)	< 0.001	< 0.001	0.001
	Stromal cells		Area	1.92 ± 0.07	1.78 ± 0.06	1.77 ± 0.05	0.180	0.055	0.875
			Intensity	2.82 ± 0.02	2.88 ± 0.04	2.92 ± 0.03	0.133	0.049	0.396
			Immunoreactivity score	5.41 ± 0.20	5.13 ± 0.19	5.23 ± 0.14	0.663	0.452	0.672
ERα	Stromal cells	Nuclear	Area	0.73 ± 0.08	1.13 ± 0.11	1.24 ± 007	< 0.001	< 0.001	0.392
			Intensity	0.65 ± 0.07	1.19 ± 0.12	1.32 ± 0.07	< 0.001	< 0.001	0.391
			Immunoreactivity score	0.85 ± 0.11	1.97 ± 0.25	2.51 ± 0.17	< 0.001	< 0.001	0.074
PR	Epithelial cells	Cytoplasmic	Area	0.82 ± 0.08	0.28 ± 0.07	0.30 ± 0.04	< 0.001	< 0.001	0.808
			Intensity	1.54 ± 0.14	0.38 ± 0.09	0.52 ± 0.06	< 0.001	< 0.001	0.197
			Immunoreactivity score	1.82 ± 0.20	0.50 ± 0.14	0.63 ± 0.08	< 0.001	< 0.001	0.455
	Stromal cells	Nuclear	Area	1.13 ± 0.06	1.56 ± 0.09	2.30 ± 0.04	< 0.001	< 0.001	< 0.001
			Intensity	2.24 ± 0.11	2.47 ± 0.09	2.94 ± 0.02	< 0.001	< 0.001	< 0.001
			Immunoreactivity score	2.85 ± 0.19	3.75 ± 0.24	6.76 ± 0.14	< 0.001	< 0.001	< 0.001
MOB4A	Epithelial cells	Cytoplasmic	Area	2.21 ± 0.13	2.06 ± 0.15	2.63 ± 0.05	< 0.001	0.002	0.001
			Intensity	1.09 ± 0.08	1.20 ± 0.11	1.49 ± 0.04	< 0.001	< 0.001	0.014
			Immunoreactivity score	2.93 ± 0.24	3.20 ± 0.31	4.09 ± 0.13	< 0.001	< 0.001	0.004
YAP1	Epithelial cells	Nuclear	Area	1.54 ± 0.09	1.27 ± 0.15	1.76 ± 0.06	< 0.001	0.048	0.002
			Intensity	1.68 ± 0.11	1.27 ± 0.15	1.78 ± 0.05	< 0.001	0.377	0.002
			Immunoreactivity score	3.03 ± 0.24	2.83 ± 0.42	3.55 ± 0.17	0.086	0.104	0.113
		Cytoplasmic	Area	1.95 ± 0.12	1.73 ± 0.14	2.42 ± 0.06	< 0.001	< 0.001	< 0.001
			Intensity	1.14 ± 0.05	1.23 ± 0.08	1.73 ± 0.11	< 0.001	< 0.001	< 0.001
			Immunoreactivity score	2.57 ± 0.26	2.90 ± 0.32	4.71 ± 0.20	< 0.001	< 0.001	< 0.001

*NNA* no neoadjuvant threatment, *NHT* neoadjuvant hormonal therapy, *CHT* neoadjuvant chemohormonal therapy, *AR* androgen receptor, *GR* glucocorticoid receptor, *ER* estrogen receptor, *PR* progesterone receptor

### Kaplan–Meier estimates of risk factors for BCR in high-risk PCa treated with CHT, followed by RP

**Fig. 5:**
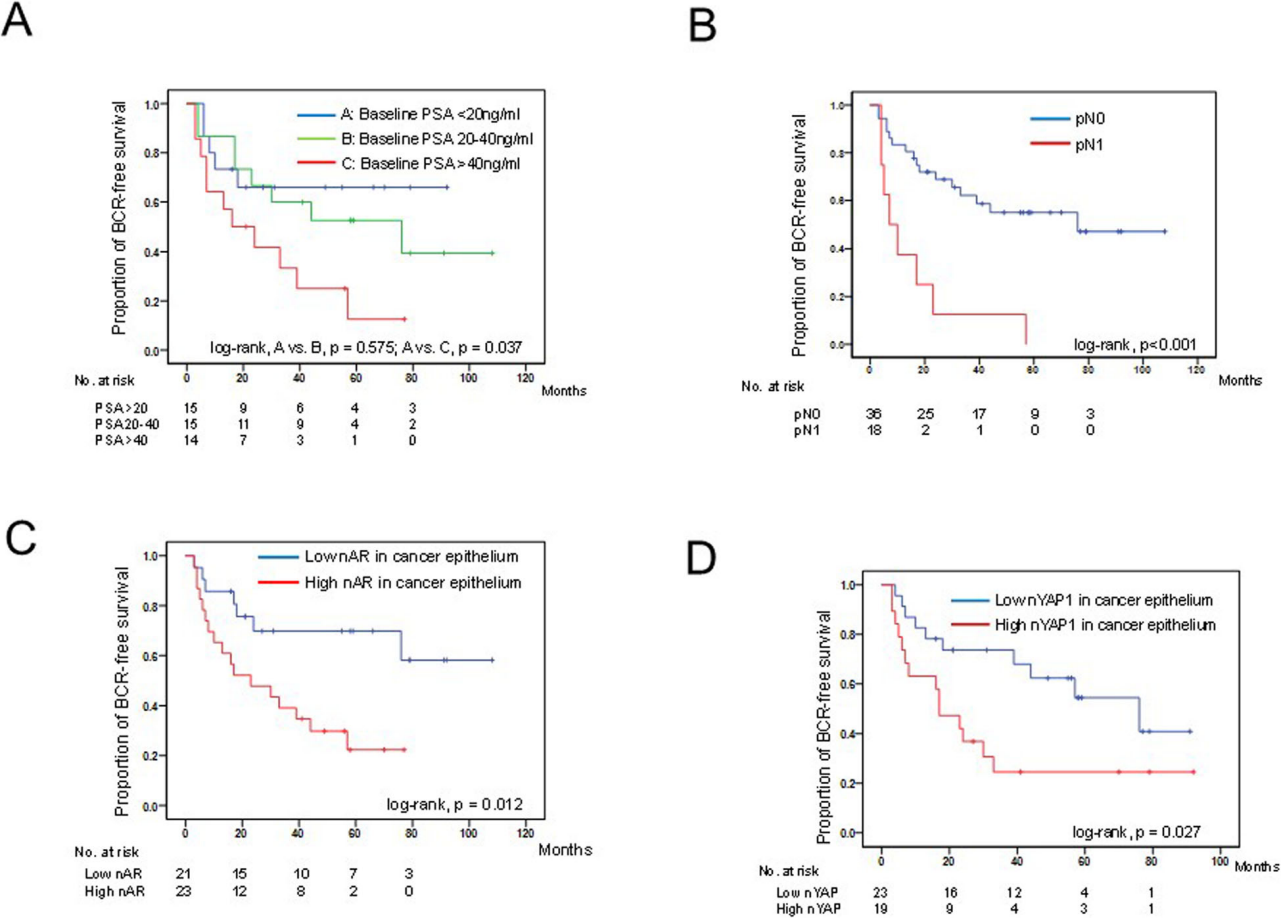
Kaplan–Meier estimates of risk factors for BCR in high-risk PCa treated with CHT, followed by RP with categorized based on the preoperative PSA level (**a**), pathological N stage (**b**), nuclear AR expression (**c**), and nuclear YAP1 expression (**d**)

To assess the impact of biomarkers for prognosis, univariate analysis of risk factors for BCR was conducted in patients with high-risk PCa treated with CHT, followed by RP. In the univariate analyses, age, baseline PSA level, pathological T stage (≥T3), pathological N (N1), nAR expression in residual cancer cells, and nYAP1 expression in residual cancer cells were significantly associated with BCR (*p* = 0.035, *p* = 0.006, *p* < 0.001, *p* = 0.001, *p* = 0.017, *p* = 0.033, respectively, Table 2, Fig. 5). Moreover, the patients with low nuclear PR expression in stromal cells tended to have a lower BCR-free survival than those with high PR expression (*p* = 0.054). On multivariable analysis, baseline PSA level, pN1, and high nYAP intensity in residual cancer cells were independent prognostic factors for BCR in patients with PCa treated with CHT, followed by RP (hazard ratio [HR] = 1.03; 95% confidence interval [CI], 1.01–1.05, *p* = 0.010; HR = 3.94; 95% CI, 1.06–14.62, *p* = 0.040; HR = 3.32; 95% CI, 1.32–8.37, *p* = 0.011; respectively, Table 2).

**Table 2:** Univariate and multivariable analysis for biochemical recurrence-free survival in patients with high-risk prostate cancer who underwent radical prostatectomy with and without neoadjuvant treatment

Variables	Univariate			Multivariable		
	HR	95%CI	*P* value	HR	95%CI	*P* value
Age	0.92	0.85–1.00	0.035			
Completion of NAC (medify vs complete)	0.84	0.35–2.02	0.689			
Baseline PSA level (continuous)	1.02	1.01–1.04	0.006	1.03	1.01–1.05	0.010
Gleason score at diagnosis (≥8 vs ≤7)	2.00	0.74–5.38	0.169			
Extended lymph node dissection (yes vs no)	1.51	0.60–3.81	0.382			
pT (≥3 vs ≤2)	4.40	1.91–10.13	< 0.001	1.13	0.30–4.22	0.854
pN (+ or 0)	4.60	1.90–11.11	0.001	3.94	1.06–14.62	0.040
Resective margin (positive or negative)	2.00	0.46–8.67	0.355			
Nuclear expression of AR in epitherial cell (High vs low)	3.00	1.22–7.35	0.017	0.98	0.32–3.02	0.971
Nuclear expression of GR in epitherial cell (High vs low)	1.82	0.79–4.18	0.161			
Nuclear expression of ERα in stromal cell (High vs low)	2.37	0.84–6.64	0.101			
Nuclear expression of PR in stromal cell (High vs low)	0.45	0.20–1.02	0.054			
Nuclear expression of YAP in epitherial cell (High vs low)	2.44	1.08–5.55	0.033	3.32	1.32–8.37	0.011
Cytoplasmic expression of MOB4A in epitherial cell (High vs low)	1.73	0.77–3.91	0.190			

*NAC* neoadjuvant treatment, *PSA* prostate specific antigen, *AR* androgen receptor, *GR* glucocorticoid receptor, *ER* estrogen receptor, *PR* progesterone receptor

## Discussion

In this study, we developed a docetaxel-resistant subline of PCa cells and showed that nYAP1 was overexpressed and highly activated in these cell lines and YAP1 knockdown in the docetaxel-resistant sublines suppressed cell proliferation in vitro*.* Furthermore, we established that TMA consisted of human PCa tissues extracted from surgery with various neoadjuvant settings. The expression of several steroid receptors and Hippo pathway–related proteins, including nYAP1, were up- and downregulated in the residual cancer and stromal cells in patients with PCa who underwent CHT compared with those treated with NNA and NHT. Notably, we found that nYAP1 expression in residual cancer cells is an independent prognostic marker for BCR in high-risk patients with PCa who underwent RP after CHT, suggesting that high levels of nYAP1 may potentially be a tissue biomarker for poor outcomes after surgery and play an important role in chemohormonal resistance in patients with PCa.

A key finding in the present study was that nYAP1 expression was strongly associated with poor BCR-free survival in patients with high-risk localized PCa treated with CHT. YAP1 was known to be associated with therapy resistance of cancer treatment [16]. A number of studies demonstrated that increased nuclear localization of YAP–TAZ and higher transcriptional activities of YAP/TAZ target genes have been observed in therapy-resistant tumors [16, 29, 30]. With regard to the relationship between YAP1 and PCa, Jiang et al. conducted a mass spectrometry–based quantitative proteomic approach and used it to compare protein phosphorylation in orthotopic xenograft tumors grown in either intact or castrated mice [31]. Their study showed that increased YAP1 levels in castration-resistant tumors and pharmacologic inhibitors of PAK2 (PF-3758309) and YAP1 (Verteporfin) inhibited the growth of androgen-independent PC3 xenografts. In a study by Zhang et al., the YAP1 mRNA was associated with androgen-insensitive PCa cells (LNCaP-C81 and LNCaP-C4–2 cells) compared with the level in androgen-sensitive LNCaP cells, and YAP1 confers castration resistance in vivo [32], strongly suggesting that YAP1 expression was associated with aggressive-phenotype PCa, particularly in the treatment-resistant stage. In the present study, we showed that YAP1 knockdown in the 22Rv1-DR cells attenuated cell proliferation without induction of apoptosis and cell cycle modulation, which indicated that future studies are needed to assess the other cellular responses in order to clarify the underlying mechanisms of YAP1-induced drug-resistance in PCa cells. Evidence on YAP1 expression in human tissues is limited; however, the rate of strong nucleus-localized YAP1 staining in resistant tumors was significantly higher than that in naive tumors in the TMA study containing naive (hormone-responsive) and castration-resistant prostate tumors [32]. In line with the results of their study, we revealed that nYAP1 was overexpressed in chemotherapy-resistant PCa.

With regard to downstream targets of YAP1 signaling, nYAP1 exerts its transcriptional activity mainly by interacting with TEADs [16] and/or Vestigial-like family member 4 (VGLL 4), which competes with YAP–TAZ for TEAD binding [33]. In the docetaxel-resistant PCa cell line, the expression of nuclear TEAD1 was assessed because several studies have shown that TEAD1 was highly expressed and correlated with poor prognosis in patients with PCa [34, 35]. However, TEAD1 expression in the nucleus of 22Rv1-DR cells was downregulated in the present study. An in vitro study using Hela cells revealed that the apoptotic role of TEAD1 was modulated by Livin, which is a family member of the inhibitor of apoptosis protein, and YAP1 was not the cofactor involved in this process [24]. The study also discussed that a modest but significant increase in Livin is observed in other types of cancer where TEAD1 is downregulated, such as breast, renal, or bladder cancer. The present study did not elucidate the details of the mechanism underlying high nYAP1 and low TEAD1 expression in docetaxel-resistant PCa cell sublines. Previous studies have described the mechanistic effects of nYAP1 on its regulators, including TEADs [36], VGLL, p160 family protein [37], and ERK-RSK signaling [32], during cancer development. Future studies to evaluate the mechanistic role of nYAP1 in prostate cancer resistance to chemotherapy using our cells and TMAs are imperative to help deepen on our understanding of these processes.

A previous study utilizing normal and cancerous human prostate tissues and PCa cell lines demonstrated that YAP1 and AR formed a protein complex in the nucleus of cancer cells under androgen-dependent and -independent conditions [17]. The study also showed that YAP1–AR interactions are androgen-independent and resistant to a novel anti-androgen, enzalutamide, in CRPC. The study further revealed that YAP1 silencing attenuated cell growth and invasion in vitro and suppressed prostate tumor xenografts in vivo. In the present study, no correlation was found between nAR and nYAP1 based on the expression level of IHC in the TMA slide (*p* = 0.228, Rs = − 0.092). However, in addition to the impact of nYAP1 on BCR, nAR was also significantly associated with BCR in the univariate analysis. The high nAR expression (activation form) and its splice variant AR-V7 have been shown to be associated with poor BCR-free survival in patients with PCa who underwent RP with and without neoadjuvant therapy [38, 39]. Therefore, the interaction between steroid receptors, including its splice variants and Hippo pathway proteins as tissue biomarkers after RP, and its orchestration on chemohormonal resistance in aggressive PCa should be investigated in future studies.

Previous studies have assessed the molecular characteristics in prostate tissues after CHT in localized PCa treated with RP [13, 14, 40, 41]. The expression analyses of selected molecular markers in patients with locally advanced or lymph node metastatic PCa treated with ADT for 1-year and three cycles of docetaxel (35 mg/m^2^ on days 1, 8, 15, and 22 every 6 weeks) showed a high nuclear expression of AR in epithelial cells of both treated and untreated patients [13]. Hence, its stromal expression increased after treatment. In contrast with that study, the expression levels of nAR in the treatment groups decreased significantly, whereas the stromal AR was overexpressed in the treatment groups in the present study. This was partly explained by the fact that the percentage of nAR positivity was immediately decreased after castration (at day 2) and gradually increased up to 120 days [42] in the animal study that evaluated nAR expression in the CWS22 human PCa xenograft after castration. Therefore, ADT duration along with the protocol of CHT may influence nAR expression after CHT, although the exact reason for the discrepancy between nAR expression in our clinical PCa specimens after CHT and nAR expression in the specimens used in the previous study was not clarified. It would be intriguing to investigate the longitudinal difference of nAR expression in the same patients with biopsy and surgical specimens at different time points after NHT and CHT.

A study that evaluated comprehensive RNA expression analyses of tissues in patients with high-risk localized PCa who underwent neoadjuvant docetaxel (36 mg/m^2^) weekly for 6 months showed no genes with large (> 5-fold) expression changes between treated and untreated prostate tumors [43]. However, a gene set composed of genes involved in androgen and estrogen metabolism was found to be coordinately upregulated in treated samples in the gene set enrichment analysis [43]. Specifically, the RNA expression of metabolic enzymes that decreased the levels of active androgen (e.g., CYP11B1, HSD11B2, HSD17B2, HSD3B1, and UGT2B15) increased, whereas that of enzymes that increased the levels of active androgens decreased (HSD11B1 and CYP11B2) [43]. These lines of evidence suggest the activation of local steroid pathways after neoadjuvant docetaxel administration. In this study, overexpression of GR was found in residual cancer cells and nuclear PR in stromal cells in the CHT group. A previous study demonstrated that acute AR inhibition resulted in GR upregulation in a subset of PCa cells due to relief of AR-mediated feedback repression of GR expression [44]. By contrast, decreased expression of the PR in cancer-associated stroma may contribute to elevated SDF-1 and interleukin-6 levels in prostate tumors and enhance prostate tumor progression, whereas the high tumor stromal cell density level (*p* = 0.045) of PR was significantly associated with tumor progression and clinical failure in tumor tissue of patients with T1-3N0 PCa undergoing RP [27, 45]. In the present study, the low stromal PR expression tended to be associated with poor BCR-free survival (HR 0.45, 95% CI, 0.20–1.02, *p* = 0.054). Although no significant impact of the expression of steroid receptors, except nAR, on BCR-free survival was found in the present study, they may potentially be associated with treatment resistance in advanced PCa.

The study using the tissue specimens collected from the multicenter phase III Cancer and Leukemia Group B 90203, which was designed to assess the impact of ADT plus docetaxel in patients with high-risk localized PCa, demonstrated the molecular analyses of pre-treatment biopsy and postoperative tissues through pathology, DNA sequence, and transcriptome profiling [40]. The study enrolled patients treated with six cycles of docetaxel at a dose of 75 mg/m^2^ administered every 3 weeks in combination with a luteinizing hormone-releasing hormone agonist for 18–24 weeks, followed by RP. With regard to the somatic mutation, the mean mutant/variant allele frequency (MAF) in post-treated RP specimens was significantly lower than that in either pre-treatment biopsy or untreated RP specimens. In the transcriptome analyses, the majority of the evaluated genes in the neoadjuvant treatment arm were significantly upregulated compared with untreated RP cancers. In terms of AR status, no AR mutation in post-treated RPs (at > 1% MAF) was detected, and overexpression of AR and AR-V7 was observed in the treated group. Although the study did not assess the expression pattern of AR using IHC, the relationship between nuclear and cytoplasmic expression patterns of AR and its splice variants in IHC and genetic/transcriptomic profiling in each patient may provide new insights into PCa aggressiveness and treatment resistance.

This study has several limitations. First, with regard to the in vitro study, only one cell line was used. Compared with well-established docetaxel-resistant prostate cancer subclones such as PC-3-DR and DU145-DR used in the previous studies [46–48], 22Rv1 has been shown to express full-length AR and its aggressive variant AR-V7 [49, 50], which reflects a real-world condition in patients with advanced prostate cancer in recent years. Nevertheless, validation using other cell lines is warranted. Second, tissues without residual mass in human tissue analyses were excluded because we focused mainly on the expression pattern of candidate biomarkers in residual prostate epithelial cells. The present study omitted the impact of stromal expression of the experimental proteins in pT0 tissues. Finally, this study did not compare the expression of each protein in tissues extracted by surgery with that in the pre-treatment biopsies. Longitudinal expression patterns before and after neoadjuvant treatment should be evaluated in a future study. Whether the expression pattern of the pre-treatment biopsies will predict the outcome of CHT still remains unknown.

## Conclusions

In conclusion, the study demonstrated that nYAP is overexpressed in docetaxel-resistant PCa cells and is a candidate prognostic marker for recurrence in patients with high-risk PCa who underwent RP, followed by CHT. Targeting Hippo pathway signaling and steroid receptors may potentially overcome chemohormonal resistance in PCa.

## Supplementary information

**Additional file 1: sTable 1.** Patient characteristics in patients with prostate cancer treated with chemohormonal therapy followed by radical prostatectomy.

**Additional file 2.** Schedule of chemohormonal therapy in patients with high-risk prostate caner in our institution.

**Additional file 3.** Panel image of TMA including prostates from the high-risk patients with localized PCa who underwent RP with or without neoadjuvant therapies.

**Additional file 4: sTable 2.** Antibodies used in the immunohistochemical analysis

**Additional file 5: sFig.3.** Original gel image of western blotting whown in Fig. 1b

**Additional file 6: sFig.4.** Original gel image of western blotting whown in Fig. 1c

**Additional file 7: sFig.5.** Original gel image of western blotting whown in Fig. 1d

**Additional file 8: sTable3.** Details of scored expression levels of steroid receptors and Hippo pathway proteins in TMA. (XLS 278 kb)

## Data Availability

The datasets used and/or analyzed during the current study are available from the corresponding author on reasonable request.

## Abbreviations

ADT: Androgen deprivation therapy
AR: Androgen receptor
BCR: Biochemical recurrence
CRPC: Castration-resistant prostate cancer
FBS: Fetal bovine serum
GR: Glucocorticoid receptor
MAF: Mutant/variant allele frequency
NHT: Neoadjuvant hormonal therapy
PR: Progesterone receptor
RP: Radical prostatectomy
