# Supplementary figures and images for: Impact of nuclear YAP1 expression in residual cancer after neoadjuvant chemohormonal therapy with docetaxel for high-risk localized prostate cancer
Source: BMC Cancer. 2020 Apr 15;20:302. doi: 10.1186/s12885-020-06844-y (PMC7333261; doi:10.1186/s12885-020-06844-y)

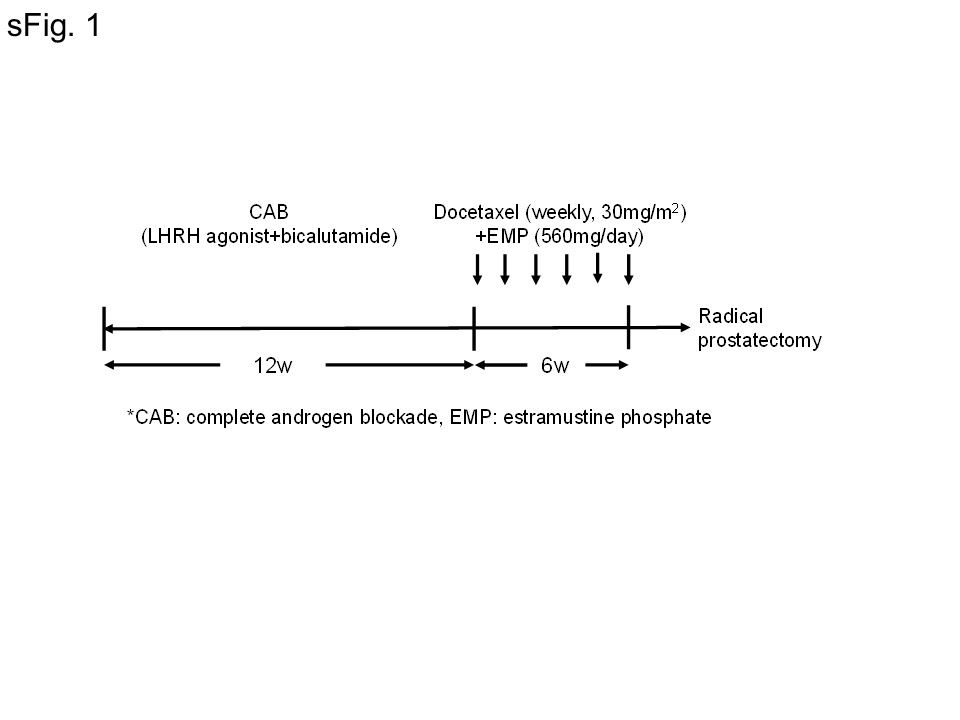

Supplement: Supplementary file 2 — Additional file 2. Schedule of chemohormonal therapy in patients with high-risk prostate caner in our institution. [file 12885_2020_6844_MOESM2_ESM.jpg]

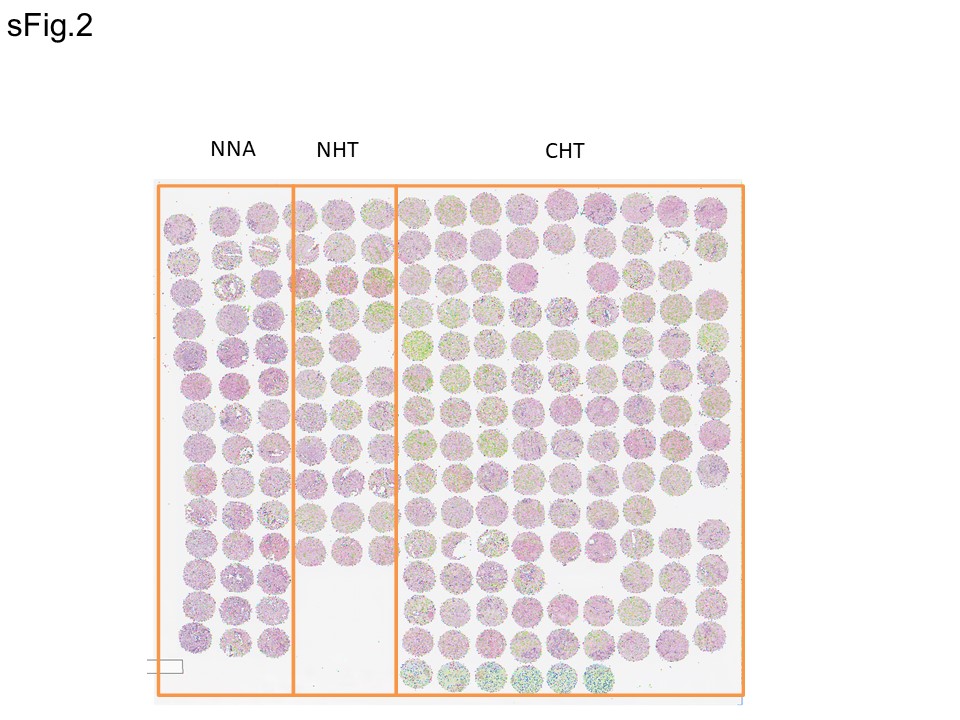

Supplement: Supplementary file 3 — Additional file 3. Panel image of TMA including prostates from the high-risk patients with localized PCa who underwent RP with or without neoadjuvant therapies. [file 12885_2020_6844_MOESM3_ESM.jpg]

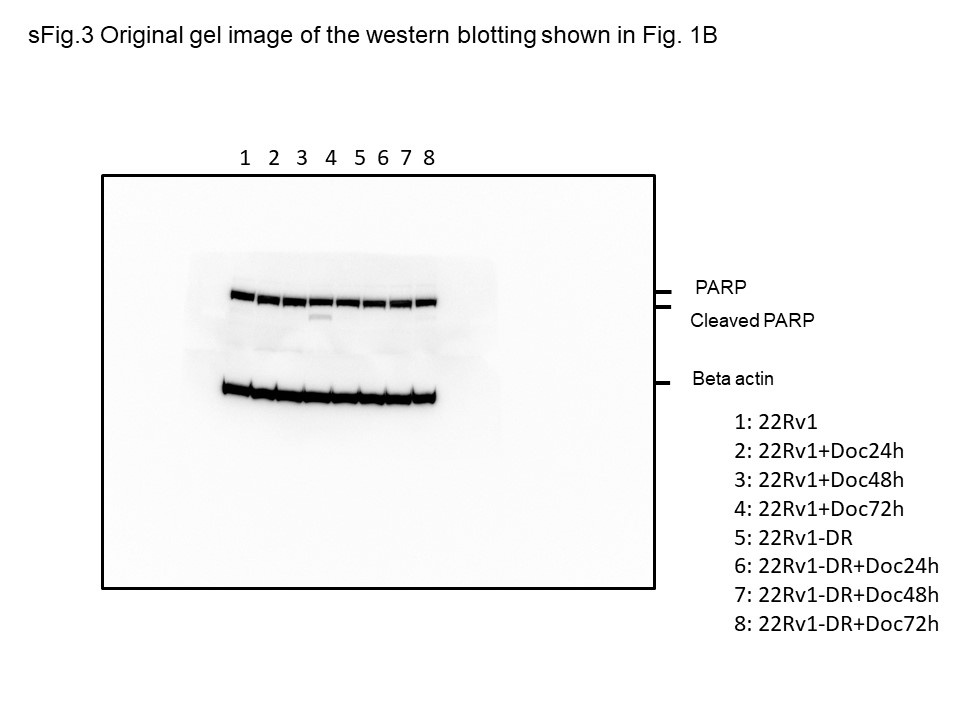

Supplement: Supplementary file 5 — Additional file 5: sFig.3. Original gel image of western blotting whown in Fig. 1b [file 12885_2020_6844_MOESM5_ESM.jpg]

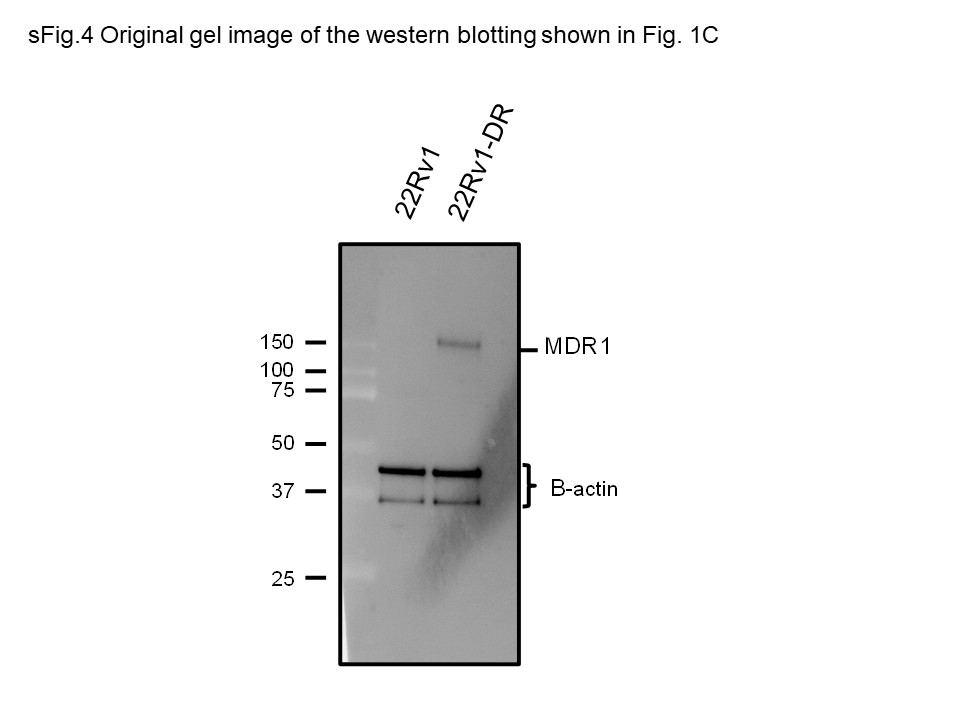

Supplement: Supplementary file 6 — Additional file 6: sFig.4. Original gel image of western blotting whown in Fig. 1c [file 12885_2020_6844_MOESM6_ESM.jpg]

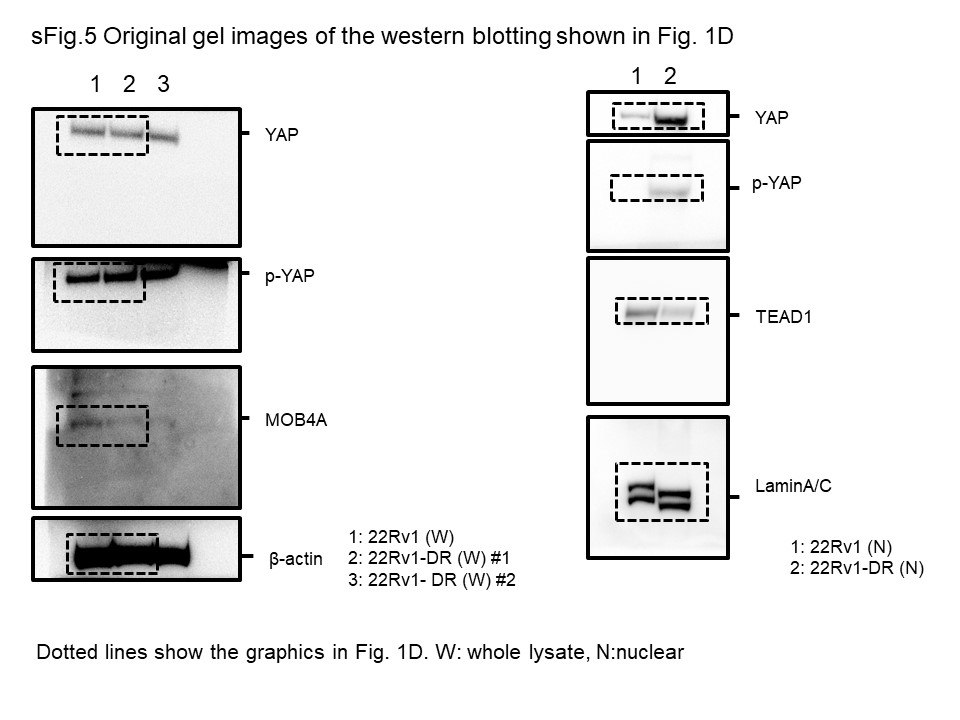

Supplement: Supplementary file 7 — Additional file 7: sFig.5. Original gel image of western blotting whown in Fig. 1d [file 12885_2020_6844_MOESM7_ESM.jpg]
